# Deflazacort: therapeutic index, relative potency and equivalent doses versus other corticosteroids

**DOI:** 10.1186/s40360-016-0111-8

**Published:** 2017-01-05

**Authors:** Luca Parente

**Affiliations:** Department of Pharmacy, University of Salerno, Fisciano, Salerno Italy

**Keywords:** Corticosteroids, Deflazacort, Equipotency ratio, Relative potencies, Therapeutic efficacy, Tolerability

## Abstract

**Background:**

Deflazacort is a synthetic corticosteroid characterized by a favourable pharmacokinetic profile, peculiar pharmacodynamic properties and a good safety profile. However, to the best of our knowledge, no dose-conversion table based on direct comparison of relative potencies between deflazacort and other main corticosteroids is currently available in scientific literature.

**Main body:**

This paper, while reporting a brief review of deflazacort pharmacological properties, its efficacy and tolerability in different clinical areas, has been designed with the specific aim of providing a new dose-conversion table of corticosteroids, including for the first time also deflazacort.

**Short conclusion:**

We suggest that this new conversion table could be a useful tool for physicians who need to select the appropriate dose of deflazacort in their clinical practice.

## Background

Corticosteroids are hormones with 21 carbon atoms; they are secreted by the adrenal cortex and are traditionally divided into two main groups: glucocorticoids and mineralocorticoids.

This distinction is based on their preferential biological activity, i.e. substances that are mainly involved in the regulation of carbohydrate metabolism (glucocorticoids) or fluid and electrolyte balance (mineralocorticoids).

The main glucocorticoid produced by the adrenal cortex is cortisol (also known as hydrocortisone); the main mineralocorticoid hormone is aldosterone.

Typically, corticosteroids are grouped according to their relative potency in terms of sodium (Na^+^) retention, their effects on carbohydrate metabolism (i.e., gluconeogenesis and glycogen deposition in the liver) and their anti-inflammatory activity.

The potency of corticosteroids on carbohydrate metabolism is closely related to their anti-inflammatory potency, as the activation of the same glucocorticoid receptor is responsible for both metabolic and anti-inflammatory effects.

On the other hand, the Na^+^ retaining potency of corticosteroids depends on activation of a different mineralocorticoid receptor; therefore, the effects on Na^+^ retention and the carbohydrate/antinflammatory actions of corticosteroids are not closely related, reflecting selective actions at distinct receptors (Table 1).

**Table 1:** Relative potencies of main corticosteroids (modified from [1])

Corticosteroid	Anti-inflammatory potency	Na^+^ retaining potency
Cortisol	1	1
Cortisone	0.8	0.8
Fludrocortisone	10	125
Prednisone	4	0.8
Prednisolone	4	0.8
6a-methylprednisolone	5	0.5
Triamcinolone	5	0
Betamethasone	25	0
Dexamethasone	25	0

The numbers in the Table indicate corticosteroid anti-inflammatory and Na^+^ retaining potency relative to cortisol (by convention, cortisol = 1), and have been calculated by the Author on the basis of currently available literature data

However, classification of corticosteroids into glucocorticoids and mineralocorticoids should not be seen as a strict rule; for example, it has been shown that corticosteroids classified in one group can also perform, in addition to their specific biological activities, certain activities that are typical of the other group, although to a lesser extent. Accordingly, some steroids predominantly classified as glucocorticoids, such as cortisol and prednisone (a synthetic corticosteroid), also have a moderate, but significant, mineralocorticoid activity; similarly, fludrocortisone, a synthetic corticosteroid with a high mineralocorticoid potency, also exhibits a moderate glucocorticoid activity [1]. Furthermore, activation of glucocorticoid receptor or mineralocorticoid receptor in the human body does not rely only on the structure of the drugs, but also on additional factors, including drug metabolism by 11- beta-hydroxydehydrogenase enzyme, which then can affect drug concentration both in the bloodstream and target tissues.

The anti-inflammatory action of corticosteroids is the main reason for their use as therapeutic agents. In its early stages, the scientific research on corticosteroids was oriented towards the synthesis of novel compounds characterized by a higher anti-inflammatory potency than cortisone, with a lower incidence of side effects.

This initially led to the development of synthetic glucocorticoids, such as prednisolone and prednisone, characterized by a higher anti-inflammatory potency than natural steroids, with a mineralocorticoid activity reduced approximately by a half.

Subsequently, other corticosteroids were synthetized, including fluorinated glucocorticoids such as dexamethasone. Even though these drugs are devoid of mineralocorticoid activity, some typical adverse events associated with their prolonged use have been reported. In fact, corticosteroid side effects often adversely affect patients’ quality of life, and safety issues are especially relevant in some populations of steroid-treated patients. For example, safety issues are relevant in children and adolescents, where steroid therapy may result in behavioral disturbances and growth retardation (due to a not yet fully understood mechanism, which probably involves abnormalities in GH release), and in elderly patients, where the risk of steroid side effects may be enhanced by the frequent presence of comorbidities requiring pharmacological polytherapy.

More recently, other corticosteroids, including deflazacort, have been synthetized with the main aim of making available new agents that are better tolerated in each age group. Deflazacort is a heterocyclic corticosteroid, oxazoline-derivative of prednisolone [2], characterized by high efficacy and good tolerability, as demonstrated by the results of several clinical studies that have evaluated its therapeutic activity and safety in conditions where corticosteroid treatment is indicated.

In particular, the structural characteristics of deflazacort can explain some of its peculiar pharmacological activities, including substantial lack of sodium-retaining activity, strong anti-inflammatory/immunosuppressive activity, and lower interference with carbohydrate metabolism and phosphocalcium metabolism (and, therefore, on growth and bone turnover) in comparison with older corticosteroids [3].

Presently, extensive clinical experience has been gained on the efficacy of deflazacort in rheumatic diseases in adults and children, as well as in respiratory, renal, and hematologic diseases. However, to the best of our knowledge, no complete dose-conversion table, based on direct comparisons of the relative potencies of deflazacort and other commonly used corticosteroids, is currently available in scientific literature. In this paper, we briefly review some of the most relevant clinical trials on deflazacort, showing its efficacy and tolerability; furthermore, we propose a new table for the conversion of corticosteroid doses, elaborated from available literature data, including both deflazacort and other common steroids used in clinical practice.

## Main text

### Deflazacort: chemical structure, mechanism of action and anti-inflammatory potency

**Fig. 1:**
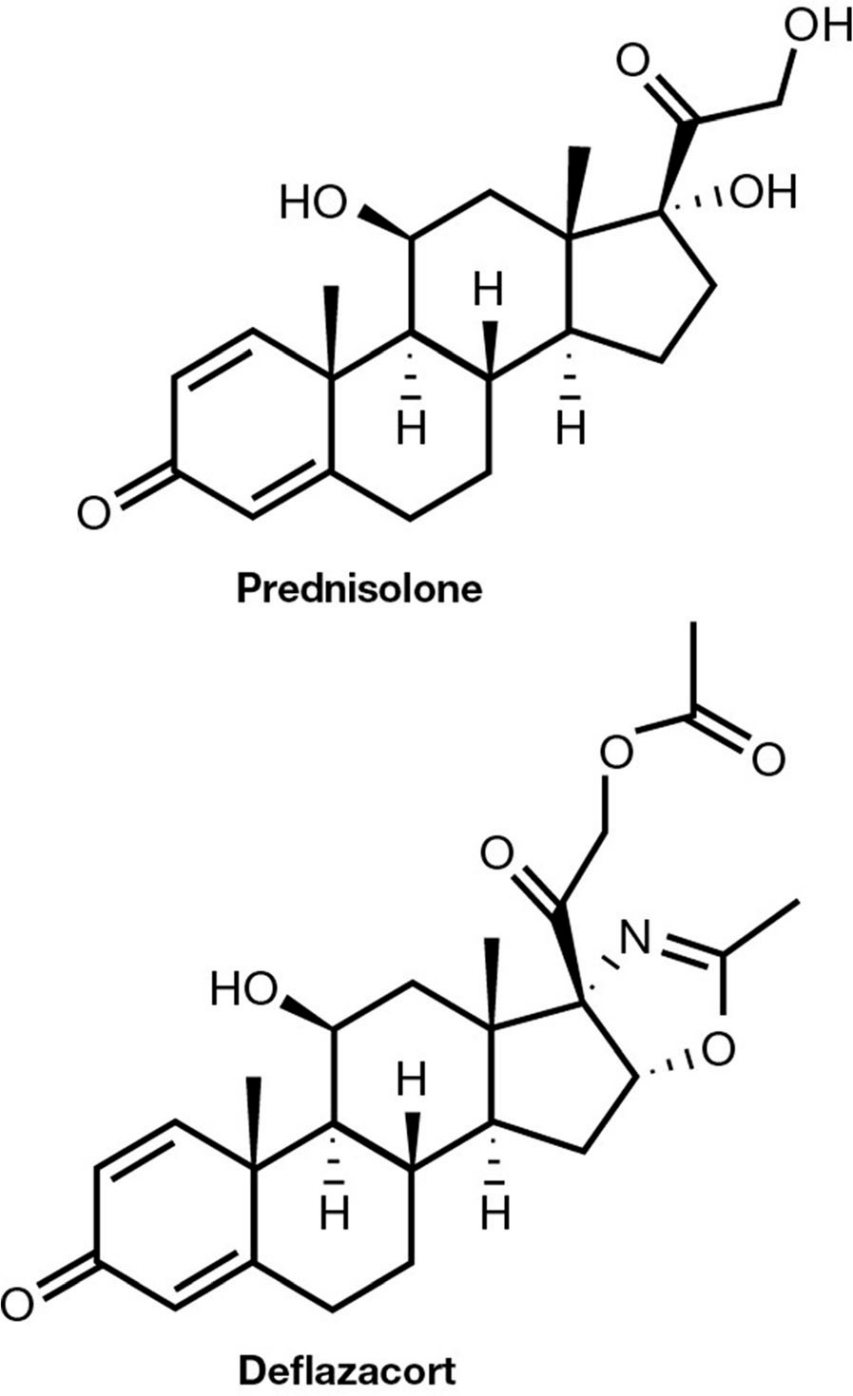
Chemical structures of prednisolone and deflazacort [3]

Deflazacort is a synthetic corticosteroid, characterized by the insertion of a methyl-oxazoline ring in the chemical structure of prednisolone 21-acetate [3] (Fig. 1).

After oral administration, deflazacort is rapidly and completely absorbed in the intestinal tract (peak plasma concentration is reached within 1–2 hours). Subsequently, deflazacort is deacetylated at position 21 to form the main active metabolite, i.e. 21-desacetyl deflazacort [4], which is characterized by a high binding affinity to tissue glucocorticoid receptors [5]. An almost complete elimination of deflazacort metabolites is reached within 24 hours, mainly through the kidneys [4].

Glucocorticoids have immunosuppressive and anti-inflammatory properties, both primarily related to the antagonism of specific leukocyte functions and to the inhibition of the synthesis of pro-inflammatory cytokines. In vitro, deflazacort significantly inhibits both the proliferation of mononuclear cells derived from human peripheral blood, and the release of inflammatory cytokines by these cells. Deflazacort is also able to inhibit chemotaxis, superoxide anion generation and chemiluminescence of human polymorphonuclear leukocytes in vitro [3].

Studies carried out using different experimental models indicate that deflazacort is an effective inhibitor of the early exudative phase of inflammation, as well as of the development of chronic granulomatous inflammation. Furthermore, deflazacort has been shown to inhibit experimentally-induced chronic inflammatory articular disease (i.e. adjuvant-induced arthritis).

Studies carried out to evaluate glucocorticoids ability to induce glycogen deposition in the liver of surrenectomized rats have shown that deflazacort is able to increase gluconeogenesis and hepatic glycogen synthesis, with a potency of action about 10 times higher than prednisolone administered at equivalent doses. The anti-inflammatory potency of deflazacort is about 10–20 times higher than prednisolone and 40 times higher than cortisol (hydrocortisone). Furthermore, the duration of action of deflazacort anti-inflammatory effects is longer than other glucocorticoids administered at equivalent doses [6].

### Deflazacort: therapeutic efficacy

Deflazacort has proven to be effective in the treatment of various rheumatological disorders including: sarcoidosis, a chronic systemic disease of unknown etiology, characterized by the formation of granulomas in various organs [7]; juvenile chronic arthritis, a condition characterized by alternating periods of remission and exacerbation, where deflazacort use resulted in clinical improvements in a group of children with active disease [8]; polymyalgia rheumatica, which occurs predominantly in elderly patients with pain and stiffness in the shoulder girdle and pelvis, where deflazacort was able to induce a significant improvement of clinical outcome parameters (pain intensity and duration of morning stiffness) and laboratory parameters, e.g. erythrocyte sedimentation rate (ESR) and C-reactive protein (CRP) [9]; rheumatoid arthritis (RA), a disease characterized by progressive, symmetrical, erosive and hypertrophic synovitis, often associated with systemic clinical involvement [10].

A clinical study, which evaluated deflazacort ability to exert a protective effect on bone erosion and joint damage, typical of RA, has shown that deflazacort is able to inhibit, in a dose-dependent manner, the invasiveness and the proliferation of synoviocytes (collected both from patients with RA and healthy volunteers) through differential modulation of single components of the fibrinolytic system [10].

Deflazacort is indicated in steroid treatment of bronchial asthma and in the exacerbations of Chronic Obstructive Pulmonary Disease (COPD), to control inflammation and increased bronchial reactivity, which are the basis of bronchospasm. Furthermore, deflazacort is useful in the treatment of children/adolescents with bronchial asthma. The efficacy and tolerability of oral deflazacort in acute moderate asthma in children has been assessed in a prospective, randomized, parallel group trial of children aged 6 to 14 years with a diagnosis of asthma, who presented to the pediatric emergency department for moderate asthma exacerbation. Short-acting ß_2_-adrenergic agonists were administered to all patients enrolled in the study; subsequently, they received either oral deflazacort (1.5 mg/kg) or prednisolone (1 mg/kg) for seven days. The primary outcome measure was Forced Expiratory Volume in one second (FEV_1_); secondary outcome measures included Peak Expiratory Flow Rate (PEFR) and pulmonary symptom score index. Patients were evaluated at the start of treatment (visit 1), on day 2 (visit 2) and on day 7 (visit 3). Two children out of the 54 enrolled were hospitalized on visit 2 (one from each group).

At baseline, clinical data (mean FEV_1_, PEFR and symptom score) were similar in both groups; on visit 2, all measures improved: FEV_1_: +22.2 and +26.5% (*p* < 0.05); PEFR: +64 and +49 L/min (*p* < 0.05); symptom score: −4.4 and −3.8 (*p* < 0.05), respectively, without significant differences between the two groups. Further improvement was observed on visit 3, i.e. FEV_1_: +33.2 and +32.0% (*p* < 0.05); PEFR: +115.7 and +87.6 L/min (*p* < 0.05); symptom score: −5.4 and −5.9 (*p* < 0.05), respectively, without significant differences between the two groups (Table 2).

**Table 2:** Results of pulmonary function tests and symptom scores recorded at baseline (visit 1) and after 2 days (visit 2) and 7 days (visit 3) of treatment with deflazacort or prednisolone in pediatric patients with moderate asthma exacerbation. Reproduced with permission from [11]

	Visit 1		Visit 2		Visit 3	
	Deflazacort	Prednisolone	Deflazacort	Prednisolone	Deflazacort	Prednisolone
FEV_1_ (%)	53 (13.3)	51 (14.9)	75* (15.8)	78* (20.3)	86*,** (14.3)	84* (18.6)
PEFR (l/min)	169.3 (65.3)	165.4 (79.3)	233* (84.7)	214 (75.5)	285* (113.4)	253* (114.8)
Symptom scores	6.1 (1.4)	6.5 (1.4)	1.7* (1.2)	2.7* (1.2)	0.7*,** (0.8)	0.6*,** (0.8)

*Significantly different (*p* < 0.05) versus visit 1; **Significantly different (*p* < 0.05) versus visit 2; FEV_1_: Forced Expiratory Volume in one second; PEFR: Peak Expiratory Flow Rate

In summary, deflazacort and prednisolone demonstrated similar efficacy in improving pulmonary function and clinical symptoms in the treatment of children with moderate exacerbations of asthma [11].

Some clinical trials, conducted both in adult and pediatric populations, have evaluated the use of deflazacort in patients undergoing renal transplantation and in patients with nephrotic syndrome.

A randomized, double-blind study compared the effects of treatment with deflazacort versus prednisone on Bone Mineral Density (BMD), on body composition and on lipid profile in 24 adult patients with end-stage renal disease, over a follow-up period of 78 months after kidney transplantation. The analysis of the 17 patients involved in the study (drop-out rates were similar in both groups) showed that lumbar BMD was significantly reduced in prednisone than in deflazacort group (*p* < 0.05); furthermore, the whole body BMD decreased from baseline in prednisone (*p* < 0.001), but not in deflazacort group. Lean body mass decreased by approximately 2.5 kg in both groups after 6–12 weeks (*p* < 0.001), then remained stable. Conversely, a significant increase in the fat mass (*p* < 0.01) and larger increases in total cholesterol (*p* < 0.03), in low-density lipoprotein cholesterol (*p* < 0.01), and in triglycerides (*p* < 0.054) were observed in the prednisone group compared to the deflazacort group. The study concluded that, in kidney-transplanted patients, the use of deflazacort is associated with lesser loss of lumbar spine BMD and whole body BMD compared to prednisone; deflazacort also helps to prevent fat accumulation and lipid profile worsening [12].

Another randomized, double-blind study compared the effects of treatment with deflazacort (0.30 ± 0.03 mg/kg per day) or methylprednisone (0.20 ± 0.03 mg/kg per day) on growth, body composition, lipid profile, and bone mass in pre-puberal patients (n = 27), during a 12-month follow-up period after kidney transplantation. The results of this study showed that the maintenance therapy with deflazacort, in comparison with methylprednisone maintenance therapy, is associated with an improved linear growth and also prevents excessive bone loss and fat accumulation in the population of pre-puberal patients [13].

Nephrotic syndrome, characterized by hypoproteinaemic edema in association with extensive proteinuria, may occur as a result of several underlying kidney diseases, the most common of which are minimal lesion glomerulonephritis and membranous glomerulonephritis. It is known that corticosteroid therapy increases the rate of spontaneous remission in patients with minimal lesion nephrotic syndrome, and has beneficial effects also in focal and segmental glomerulosclerosis.

Clinical trials have demonstrated the efficacy of deflazacort treatment in both adult and pediatric patients with nephrotic syndrome. Two clinical trials found that deflazacort was at least as effective as prednisone in adult patients with idiopathic nephrotic syndrome (during a 3-month crossover study) as well as in patients with minimal change, membranoproliferative, focal segmental or membranous glomerulonephritis (during a 12-month double-blind parallel study) [3].

A clinical complication of nephrotic syndrome is hypogammaglobulinemia (due to increased catabolism and urinary loss of immunoglobulin) which is associated with a higher risk of infections, mainly caused by encapsulated bacteria. In this respect, some evidence indicate that IgG2-subclass antibodies are protective against this type of microorganisms, so that their loss in nephrotic syndrome is a risk factor for infection.

A study on patients with nephrotic syndrome (*n* = 11; mean age 48 months, range 16–52 months) evaluated the efficacy of deflazacort versus methylprednisolone in reducing the loss of the different immunoglobulin subclasses, during periods of both remission and relapse. In both treatment groups, a similar return to normal levels of total IgG and IgG1 was observed, but only those patients treated with deflazacort showed a significant improvement in the IgG2 and IgG3 subclasses values [14].

### Deflazacort: tolerability

Deflazacort has a smaller impact on calcium metabolism than any other synthetic corticosteroid, and therefore shows a lower risk of growth rate retardation in children [15, 16] and of osteoporosis in adult/elderly patients [2]. Another distinctive feature of deflazacort is its smaller influence on carbohydrate metabolism than other glucocorticoids, as demonstrated in both studies conducted on animal models [4] and in clinical trials [17, 18]. Furthermore, the risk of sodium retention and hypokalemia, related to the mineralocorticoid activity of the drug, is greatly reduced with deflazacort in comparison with older synthetic steroids [3].

Because of its low liposolubility, only a small part of 21-desacetyl-deflazacort, (biologically active metabolite of deflazacort), crosses the blood–brain barrier [5]. Thus, when compared with older corticosteroids, deflazacort has a smaller suppressive effect on hypothalamic-pituitary-adrenal (HPA) axis, a system that modulate the physiologic response to stressors and regulate glucose and phosphocalcium metabolism and electrolyte balance.

Furthermore, given the minor impact of deflazacort on the HPA axis, a reduced risk of behavioral disorders compared to other corticosteroids has been hypothesized.

Deflazacort safety in children is a clinically relevant issue. In fact, when using corticosteroids in pediatric patients, and especially after long-term treatment, one of the most alarming adverse event is linear growth retardation; such event could be due to several mechanisms, the main of which seems to be the inhibition of Growth Hormone (GH) release from the pituitary gland. In children receiving long-term steroid treatment after kidney transplantation, the substitution of methylprednisolone (mean dose, 0.2 mg/kg per day) with deflazacort (mean dose, 0.3 mg/kg per day), over an average period of 15 months, significantly increased GH secretion (*p* < 0.05) and mean growth velocity (*p* < 0.01) [13].

### Deflazacort: therapeutic indications and dosage

Deflazacort therapeutic indications include a number of pathological conditions, i.e.: primary and secondary *adrenocortical failure*; *rheumatic diseases* (including RA and ankylosing spondylitis); *collagen diseases* (such as systemic lupus erythematosus and polymyositis); *dermatological diseases* (including pemphigus, severe psoriasis and mycosis fungoides); *allergic states* (e.g., bronchial asthma and drugs hyper-reactivity); *respiratory diseases* (including sarcoidosis and aspiration pneumonia); *ocular diseases* (e.g., chorioretinitis and optic neuritis); *hematological diseases* (including Hodgkin's disease, non-Hodgkin's lymphoma, chronic lymphocytic leukemia and acute childhood leukemia); *edematous state* (nephrotic syndrome); *gastro-intestinal diseases* (ulcerative colitis, regional enteritis).

The initial daily dosage of deflazacort in adults may range from 6 to 90 mg, depending on the severity and evolution of the specific disease, and should remain unchanged, or be modified as appropriate, until a satisfactory clinical response is observed. It should be emphasized that corticosteroid requirements vary from patient to patient, so the dosage should be individualized taking into account the nature of the disease and the patient's therapeutic response.The maintenance dose should always be the minimum dose required to control the symptoms in order to minimize the risk of side effects; in any case, the dose should be reduced gradually [6].

#### Relative potencies and equivalent doses of corticosteroids: general considerations

Corticosteroids were initially introduced in clinical practice in the late '40s, when they began to be used for the treatment of rheumatoid arthritis; since then, corticosteroid therapeutic indications have expanded to various medical areas, including dermatology, rheumatology, immunology and oncology. Systemic corticosteroids, which are used to treat several pathological conditions, are similar to endogenous steroid hormones produced by the adrenal cortex, i.e. mineralocorticoid (e.g. aldosterone) and glucocorticoids (e.g. cortisol).

Mineralocorticoid are mainly regulated by the renin-angiotensin system and increase Na^+^ retention; glucocorticoids are mainly regulated by Adrenocorticotropic Hormone (ACTH) and can exert anti-inflammatory, as well as metabolic and immunologic effects.

Although all corticosteroids share, to varying degrees, the properties of both adrenal hormones, some of them (e.g. fludrocortisone) are essentially used because of their mineralocorticoid activity, while others (e.g. hydrocortisone, cortisone, prednisone and prednisolone) are primarily used for their glucocorticoid effects [19].

The precise knowledge of the characteristics and physiological effects of corticosteroids is essential to select the most appropriate drug and to avoid problems due to its inappropriate use; therefore, the equipotency ratios between steroids is an important aspect to be considered in long-term steroid therapy.

The anti-inflammatory potency of cortisol (hydrocortisone) is conventionally equal to 1; hence, based on the relative potencies of various corticosteroids, it is possible to identify the equivalent doses of every single steroid.

Table 3 summarizes the relative potencies and the corresponding equivalent doses of systemic corticosteroids according to available literature data [20] (see next paragraph for deflazacort, as it is not included in this Table).

**Table 3:** Relative potencies and equivalent doses of corticosteroids [Modified from 20]

	Equivalent glucocorticoid dose (mg)	Potency relative to hydrocortisone (cortisol) (antiiflammatory activity)	Potency relative to hydrocortisone (cortisol) (mineralcorticoid activity)
Short acting			
Hydrocortisone (cortisol)	20	1	1
Cortisone acetate	25	0.8	0.8
Intermediate acting			
Prednisone	5	4	0.8
Prednisolone	5	4	0.8
Triamcinolone	4	5	N/A
Methylprednisolone	4	5	0.5
Long acting			
Dexamethasone	0.75	30	N/A
Betamethasone	0.6	30	N/A
Mineralcorticoids			
Fludrocortisone	N/A	15	150
Aldosterone	N/A	N/A	400

The numbers in the table indicate corticosteroid potency and equivalent doses relative to cortisol (by convention, cortisol = 1), and have been calculated by the Author on the basis of currently available literature data. N/A, not applicable

*Short-acting* compounds, such as hydrocortisone (cortisol), show lower potencies, while *intermediate-acting* drugs, like prednisone and methylprednisolone, are four to five times more potent than hydrocortisone; dexamethasone, a *long-acting* corticosteroid, shows a potency about 25 times higher than short-acting compounds. Corticosteroids therapeutic effects may often be associated with side effects, especially when higher doses and long-term treatment are required. To obtain the maximum benefit from therapy, it is important to prevent any potential adverse event by implementing appropriate preventive measures, as far as possible [19].

However, it should be emphasized that the risk of side effects is not only related to corticosteroid dose and duration of treatment; it also depends on the so-called Drug Therapeutic Index (DTI), i.e. a ratio that compares the blood concentration at which a drug becomes toxic to the concentration at which the drug exerts the desired therapeutic effect. The drug concentration that produces a specific effect in a patient is indicated as “individual effective concentration”; the pharmacological response, in this case, is defined as "quantal", since the specific effect may - or may not- occur.

The dose of a drug required to produce a specified “quantal effect” in 50% of the individuals is the “median effective dose”, commonly indicated as Effective Dose-50 (ED_50_). In preclinical studies, the drug “median lethal dose”, determined on experimental models, is defined as Lethal Dose-50 (LD_50_); therefore, in experimental models, the ratio between LD_50_ and ED_50_ indicates DTI.

In clinical studies, the therapeutic index can be determined by comparing the dose, or the drug concentration required to cause toxicity, with the concentration required to produce the therapeutic effects in the studied population. However, it should be considered that “in vivo” no drug produces only a single effect, so the therapeutic index of a drug may be variable depending on the specific effect that is measured. However, a wider therapeutic index usually indicates a lower number and severity of side effects associated with the use of the same drug [1].

## Relative potencies and equivalent doses: deflazacort versus other corticosteroids

Corticosteroid doses used in clinical practice may be highly variable, depending on the disease to be treated and its severity. The reference glucocorticoid is prednisone, usually administered at a dose ranging from 5 to 60 mg/day. Deflazacort is less potent than prednisone and is usually administered at proportionately higher doses.

Based on the results of seven clinical trials enrolling a total of 160 patients, deflazacort is approximately 25% less potent than prednisone in terms of absolute dosage. The potency ratio of prednisone to deflazacort is about 1:1.3; e.g., 8 mg of deflazacort elicits a similar biological response to 6 mg of prednisone.

The dose equivalence of orally administered deflazacort and prednisone has been evaluated in a large US trial. In this study, patients with corticosteroid-dependent asthma or asthmatic bronchitis were treated with prednisone 40 mg/day for a 2 week run-in period, in order to establish a high baseline corticosteroid dosage level. After this run-in period, patients were randomised to receive deflazacort (initial dosage 36 mg/day) or prednisone (initial dosage 30 mg/day); the dose of each drug was progressively reduced until the minimum effective dose was reached. The minimum effective dose was deemed to have been reached when a minimum of two of the following events was reported: asthma-related awakenings on 2 of 7 consecutive nights; ≥20% decrease in morning PEFR (versus prior evening value) on 4 of 7 consecutive mornings; the sum of patient-assessed symptom scores for 7 consecutive days was lower than that for the final 7 days of the run-in period. On this basis, the minimum effective dose of deflazacort was 27.2 mg/day (*n* = 45) compared with 20.5 mg/day for prednisone (*n* = 46), i.e. a ratio of 1.3:1 [3].

The precise determination of the equipotency ratio of deflazacort versus other glucocorticoids is an important safety issue with regard to the long-term therapy with these drugs; for example, glucocorticoid-induced osteoporosis appears to be dose-dependent and is documented for prednisolone-equivalent doses ≥5 mg/day.

With regard to the potential of drug-induced osteoporosis, an equipotency ratio of deflazacort versus prednisolone of 1.2:1 was considered probable in previous studies; however, more recent data indicate that the correct equipotency ratio deflazacort/prednisolone may be at least 1.5:1, and deflazacort/methylprednisolone 1.875:1. Consequently, at standard doses, the osteoporotic potential of deflazacort appears to be much lower than other steroids.

A clinical study that enrolled 21 male patients with active RA or non-axial Psoriatic Arthropathy (PsA), naïve to steroid treatment, reported useful information to establish a correct dose-equivalence ratio between low-dose deflazacort and methylprednisolone [21]. The patients enrolled in this study were randomized in two groups: 10 patients were treated for 6 months with deflazacort 7.5 mg/day and calcium, cholecalciferol and a Disease-Modifying Antirheumatic Drug (DMARD) (Group 1); 11 patients were treated for 6 months with methylprednisolone 4 mg/day, calcium, cholecalciferol and a DMARD, and for the following 6 months with deflazacort 7.5 mg/day, calcium, cholecalciferol and a DMARD (Group 2).

The efficacy of treatment was evaluated in each group at day 0, 90, 180, 240, and 360. Clinical efficacy parameters included: number of swollen joints; number of tender joints; pain measured by means of a Visual Analogue Scale (VAS); global self-assessment of efficacy expressed separately by the physician and the patient using a VAS; disability index calculated by means of the Health Assessment Questionnaire (HAQ). Similar trends of efficacy were observed in both groups, with significant improvements of clinical parameters already evident after 3 months of treatment; these improvements were maintained throughout the treatment period, regardless of the corticosteroid used. The study conclusion was that deflazacort is effective in the treatment of inflammatory arthropathies in male adults, with a deflazacort/methylprednisolone equivalence ratio of 1.875:1, and a deflazacort/prednisolone equivalence ratio of 1.5:1 [21].

Other studies, however, suggest that deflazacort/prednisolone equivalence ratio may depend on the disease, e.g. approximately 1.2:1 in rheumatoid arthritis, juvenile chronic arthritis and nephrotic syndrome, and approximately 1.4:1 in asthma and polymyalgia rheumatica [22]. However, although several studies have determined the equipotency ratio of deflazacort versus other corticosteroids, to the best of our knowledge, no dose-conversion table that includes also deflazacort is so far available in the literature. Therefore, to facilitate identification of the correct dosage of this drug in clinical practice, we propose here a novel conversion table of corticosteroids doses, elaborated from literature available data (i.e. clinical trials with sufficiently homogenous study populations) which also includes deflazacort [1, 3, 19–21] (Table 4).

**Table 4:** Conversion of corticosteroid doses. Elaboration of data from [1] and [3]

Corticosteroid	Approximate equivalent doses (mg)
Cortisone [1]	25
Hydrocortisone [1]	20
Deflazacort [3]	7.5
Prednisolone [1]	5
Prednisone [1]	5
Methylprednisolone [1]	4
Triamcinolone [1]	4
Betamethasone [1]	0.75
Dexamethasone [1]	0.75

## Conclusion

The anti-inflammatory effect of corticosteroids is one of the main reasons for the use of these therapeutic agents. Several studies on adrenal steroids with glucocorticoid activity have led to the synthesis of new molecules characterized by high anti-inflammatory potency and low incidence of side effects. Safety issues are particularly relevant in some populations on long-term therapy with corticosteroids, such as children and adolescents, in whom steroid therapy may result in growth retardation and behavioral disorders, as well as in somewhat "fragile" individuals such as politreated elderly patients.

Deflazacort is a recent synthetic glucocorticoid characterized by high efficacy and good tolerability, as demonstrated by several clinical studies that have evaluated its therapeutic efficacy and safety of use. In comparison to older corticosteroids, deflazacort shows a high anti-inflammatory and immunosuppressive activity, and a lower interference on glucose metabolism, on phosphocalcium metabolism (hence, on growth and bone turnover), and on HPA axis functionality, as well as a minimal or absent sodium-retention effect.

These characteristics are favourable and suggest that deflazacort can be used safely even in pediatric and elderly populations. Furthermore, deflazacort shows a high dosage flexibility, due to its wide therapeutic index; in fact, the initial oral daily dosage of deflazacort in adults ranges from 6 to 90 mg, depending on the severity and progression of the specific disease to be treated.

The precise knowledge of the characteristics and physiological effects of corticosteroids can assist in the correct choice of the drug, avoiding potential problems due to its inappropriate use. Therefore, the exact quantification of the equipotency ratios between steroids is an important aspect to consider when using these drugs for long periods of time.

At present there are few (if any) clinical reasons for preferentially using any steroid drug and therefore clinical experience, bioavailability and bio-pharmaceutical factors clearly affect choice of steroid for a given medical condition; however, knowing the relative potencies and equivalent doses of corticosteroids in clinical practice is useful.

We suggest that the new table for the conversion of corticosteroid doses presented in this paper, which for the first time includes deflazacort, could provide an aid to physicians when selecting the appropriate dosage of corticosteroids to be used in clinical practice.

The evaluation of deflazacort dose range and its therapeutic effectiveness in comparison with other corticosteroids could be improved by a specific meta-analysis; indeed, we hope that this paper could stimulate other Authors to conduct a meta-analysis on these topics.

## Abbreviations

ACTH: Adrenocorticotropic hormone
BMD: Bone Mineral Density
COPD: Chronic Obstructive Pulmonary Disease
CRP: C-Reactive Protein
DMARD: Disease-Modifying Antirheumatic Drug
DTI: Drug Therapeutic Index
ED_50_: Effective Dose-50
ESR: Erythrocyte Sedimentation Rate
FEV_1_: Forced Expiratory Volume in one second
GH: Growth Hormone
HAQ: Health Assessment Questionnaire
HPA: Hypothalamic-pituitary-adrenal
IgG: Immunoglobulin G-type
LD_50_: Lethal Dose-50
N/A: Not applicable
PEFR: Peak Expiratory Flow Rate
PsA: Psoriatic Arthropathy
RA: Rheumatoid Arthritis
VAS: Visual Analogue Scale
